# Ataxie spino-cérébelleuse de type 7 (SCA) dignostiqué par l'aspect du fond d’œil

**DOI:** 10.11604/pamj.2014.17.110.3961

**Published:** 2014-02-14

**Authors:** Zineb Jaja, Amina Laghmari

**Affiliations:** 1 Université Mohammed V Souissi, Service d'Ophtalmologie A de l'hôpital des Spécialités, Centre Hospitalier Universitaire, Rabat, Maroc

**Keywords:** Ataxie spino-cérébelleuse de type 7, fond d’œil, maculopathie atrophique, Type 7 spinocerebellar ataxia, fondus, atrophic maculopathy

## Image en medicine

Les ataxies spinocérébelleuses constituent un groupe de maladies neurodégénératives très hétérogènes tant du point de vue clinique que génétique, d’évolution lente et progressive. L'ataxie spino-cérébelleuse de type 7 (SCA7) est un type d'ataxie associé à une dégénérescence rétinienne. Nous présentons un cas familial de SCA7 avec maculopathie atrophique bilatérale. Il s'agit d'un patient âgé de 24 ans présentant depuis l'adolescence une baisse de l'acuité visuelle progressive associée à une ataxie et une dysarthrie. L'interrogatoire a permis de déceler la présence de deux cas similaires dans sa famille. A l'examen, on trouve au niveau des deux yeux : une AV réduite au décompte les doigts de près, un segment antérieur normal et au FO une aire d'atrophie maculaire circulaire. L'ERG global et P-ERG ont montré des réponses altérées des deux systèmes photopique et scotopique. L'examen de la vision colorée a montré une dyschromatopsie sans axe précis. L'examen neurologique et la neuro-imagerie étaient en faveur d'une affection hérédo-dégénérative type SCA7. La SCA7 appartient aux ataxiescérébelleusesautosomiques dominantesde type II (ACAD II), selon la classification de Harding. Ellese caractérise par une ataxie cérébelleuse progressiveet et une dystrophie rétinienne progressive notamment maculaire, avec perte de la vision centrale entraînant la cécité chez les adultes touchés. L'atteinte rétinienne permet de classer cette forme clinique. Le bilan électrophysiologique demandé devant la baisse d'acuité visuelle permet de préciser le type de dysfonctionnement rétinien, tel qu'il a été le cas chez notre patient. L'examen ophtalmologique avec électrophysiologie rétinienne et l'examen neurologique avec exploration neuroradiologique permettent de poser le diagnostic.

**Figure 1:**
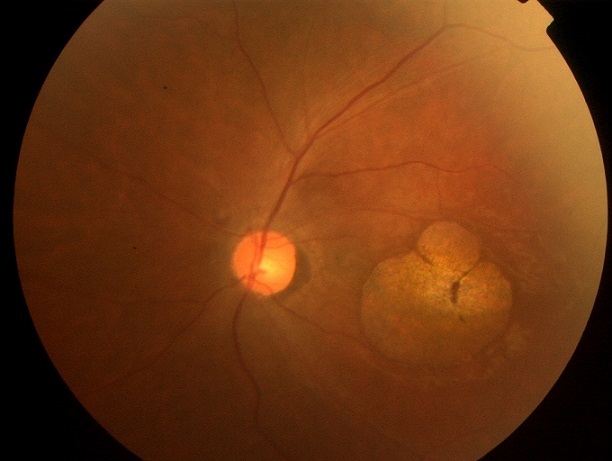
Atrophie maculaire circulaire olycyclique bilatérale

